# 5,5′-Bis(diethyl­amino)-2,2′-[butane-1,4-diyldioxy­bis(nitrilo­methyl­idyne)]­diphenol

**DOI:** 10.1107/S1600536808004352

**Published:** 2008-03-05

**Authors:** Gai-Lan Liu, Xiao Chen, Xue-Ni He, Wen-Kui Dong

**Affiliations:** a School of Chemical and Biological Engineering, Lanzhou Jiaotong University, Lanzhou 730070, People’s Republic of China

## Abstract

The title complex, C_26_H_38_N_4_O_4_, was synthesized by the reaction of 4-diethyl­amino-2-hydroxy­benzaldehyde with 1,4-bis­(amino­oxy)butane in ethanol. It crystallizes as discrete centrosymmetric molecules adopting an extended conformation where the two salicylaldoxime groups are separated from each other. Intra­molecular O—H⋯N hydrogen bonding is observed between the hydr­oxy groups and oxime N atoms. Inter­molecular π–π stacking inter­actions [3.979 (2) Å] between aromatic rings are apparent in the crystal structure. Each ethyl group is disordered over two positions; in one the site occupancy factors are 0.55 and 0.45, in the other 0.53 and 0.47.

## Related literature

For related literature, see: Abu-Surrah *et al.* (1999 ▶); Boghaei *et al.* (2006 ▶); Costes *et al.* (2000 ▶); Dong, Duan *et al.* (2007 ▶); Dong, He *et al.* (2007 ▶); Lacroix (2001 ▶); Zhang *et al.* (2007 ▶).
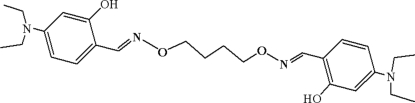


## Experimental

### 

#### Crystal data

C_26_H_38_N_4_O_4_
*M* *_r_* = 470.60
Monoclinic, 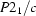

*a* = 7.6888 (9) Å
*b* = 13.777 (2) Å
*c* = 12.6547 (19) Å
β = 101.627 (2)°
*V* = 1313.0 (3) Å^3^
*Z* = 2
Mo *K*α radiation
μ = 0.08 mm^−1^
*T* = 298 (2) K
0.45 × 0.43 × 0.37 mm

#### Data collection

Bruker SMART CCD area-detector diffractometer
Absorption correction: multi-scan (*SADABS*; Sheldrick, 1996 ▶) *T*_min_ = 0.965, *T*_max_ = 0.971
6450 measured reflections
2303 independent reflections
1176 reflections with *I* > 2σ(*I*)
*R*_int_ = 0.034

#### Refinement

*R*[*F* ^2^ > 2σ(*F* ^2^)] = 0.051
*wR*(*F* ^2^) = 0.152
*S* = 1.06
2303 reflections
196 parameters
H-atom parameters constrained
Δρ_max_ = 0.12 e Å^−3^
Δρ_min_ = −0.15 e Å^−3^

### 

Data collection: *SMART* (Siemens, 1996 ▶); cell refinement: *SAINT* (Siemens, 1996 ▶); data reduction: *SAINT*; program(s) used to solve structure: *SHELXS97* (Sheldrick, 2008 ▶); program(s) used to refine structure: *SHELXL97* (Sheldrick, 2008 ▶); molecular graphics: *SHELXTL* (Sheldrick, 2008 ▶); software used to prepare material for publication: *SHELXTL*.

## Supplementary Material

Crystal structure: contains datablocks global, I. DOI: 10.1107/S1600536808004352/hg2359sup1.cif

Structure factors: contains datablocks I. DOI: 10.1107/S1600536808004352/hg2359Isup2.hkl

Additional supplementary materials:  crystallographic information; 3D view; checkCIF report

## Figures and Tables

**Table 1:** Hydrogen-bond geometry (Å, °)

*D*—H⋯*A*	*D*—H	H⋯*A*	*D*⋯*A*	*D*—H⋯*A*
O2—H2⋯N1	0.82	1.91	2.639 (2)	147

**Fig. 1.**
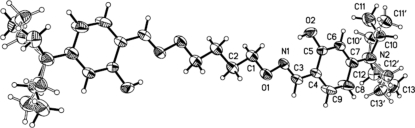
The molecule structure of (I) with atom labelling and displacement ellipsoids at the 30% probability level for non-hydrogen atoms.

## supplementary crystallographic information

### Comment

A great deal of attention has recently been attracted to the study of salen and its derivatives (Boghaei *et al.*, 2006; Abu-Surrah *et al.*, 1999) due to the ease of formation of metal complexes which model reaction centers of metalloenzymes. These compounds also have excellent magnetic properties (Costes *et al.*, 2000) and form nonlinear optical materials (Lacroix, 2001). Recently, we have reported some salen-type bisoxime derivatives (Dong, Duan *et al.*, 2007; Dong, He *et al.*, 2007; Zhang *et al.*, 2007), Now, the title compound, (I) was synthesized and its crystal structure determined. (Fig. 1). The molecule of (I) is disposed about a crystallographic centre of symmetry, and adopts an extended conformation with the two salicylaldoxime groups separated from each other.

The oxime groups and phenolic groups adopt a *trans* conformation about the C?N bond, and there is a strong O—H···N intramolecular hydrogen bond, O2—H2···N1 (*d*(O2—H2) = 0.820 Å, d(H2···N1) = 1.913 Å, d(O2···N1) = 2.639 (2) Å, <O2—H2···N1 = 147.01°). The carbon atoms of *N*,*N*'-diethylamino of the ligands (C10, C11, C12, C13 and C10', C11', C12', C13') are disordered over two different positions, which were allowed for during refinement.

### Experimental

5,5'-di(*N*,*N*'-diethylamino)-2,2'-[(1,4-butylene) dioxybis(nitrilomethylidyne)]diphenol was synthesized according to an method reported earlier (Zhang *et al.*, 2007). To an ethanol solution (5 ml) of 4-(*N*,*N*-diethylamino)-2-hydroxybenzaldehyde (398.07 mg, 2.06 mmol) was added an ethanol (3 ml) solution of 1,4-bis(aminooxy)butane (121.66 mg, 1.03 mmol). The solution was stirred at 328 K for 4 h. then concentrated to about 2 ml under reduced pressure, and washed successively with ethanol and hexane, respectively. The product was dried under vacuum and purified with recrystallization from ethanol to yield 436.26 mg of the title compound. Yield, 45%. mp. 397–398 K. Anal. Calc. for C_26_H_38_N_4_O_4_: C, 66.36; H, 8.14; N, 11.91%. Found: C, 66.25; H, 8.08; N, 12.07%. Colorless prismatic single crystals suitable for X-ray diffraction studies were obtained after about two weeks by slow evaporation of (I) at room temperature from an acetone/chloroform solution

### Refinement

H atoms were treated as riding atoms with distances C—H = 0.97 (CH_2_), or 0.93 Å (CH), O—H = 0.82 Å, and *U*_iso_(H) = 1.2 *U*_eq_(C) and 1.5 *U*_eq_(O). The hydroxyl protons were located directly from a Fourier difference map. Each ethyl group is disordered over two positions; in one the site occupancy factors are 0.55 and 0.45, in the other 0.53 and 0.47.

### Crystal data

**Table:**

C_26_H_38_N_4_O_4_	*F*_000_ = 508
*M**_r_* = 470.60	*D*_x_ = 1.190 Mg m^−^^3^
Monoclinic, *P*2_1_/*c*	Mo *K*α radiation λ = 0.71073 Å
Hall symbol: -P 2ybc	Cell parameters from 1484 reflections
*a* = 7.6888 (9) Å	θ = 3.0–23.4º
*b* = 13.777 (2) Å	µ = 0.08 mm^−^^1^
*c* = 12.6547 (19) Å	*T* = 298 (2) K
β = 101.627 (2)º	Block, colourless
*V* = 1313.0 (3) Å^3^	0.45 × 0.43 × 0.37 mm
*Z* = 2	

### Data collection

**Table:**

Bruker SMART CCD area-detector diffractometer	2303 independent reflections
Radiation source: fine-focus sealed tube	1176 reflections with *I* > 2σ(*I*)
Monochromator: graphite	*R*_int_ = 0.034
*T* = 298(2) K	θ_max_ = 25.0º
φ and ω scans	θ_min_ = 2.2º
Absorption correction: multi-scan(SADABS; Sheldrick, 1996)	*h* = −9→8
*T*_min_ = 0.965, *T*_max_ = 0.971	*k* = −16→16
6450 measured reflections	*l* = −15→14

### Refinement

**Table:**

Refinement on *F*^2^	Secondary atom site location: difference Fourier map
Least-squares matrix: full	Hydrogen site location: inferred from neighbouring sites
*R*[*F*^2^ > 2σ(*F*^2^)] = 0.051	H-atom parameters constrained
*wR*(*F*^2^) = 0.152	*w* = 1/[σ^2^(*F*_o_^2^) + (0.0421*P*)^2^ + 0.5356*P*] where *P* = (*F*_o_^2^ + 2*F*_c_^2^)/3
*S* = 1.06	(Δ/σ)_max_ = 0.001
2303 reflections	Δρ_max_ = 0.12 e Å^−^^3^
196 parameters	Δρ_min_ = −0.15 e Å^−^^3^
Primary atom site location: structure-invariant direct methods	Extinction correction: none

### Special details

**Table:**

Geometry. All e.s.d.'s (except the e.s.d. in the dihedral angle between two l.s. planes) are estimated using the full covariance matrix. The cell e.s.d.'s are taken into account individually in the estimation of e.s.d.'s in distances, angles and torsion angles; correlations between e.s.d.'s in cell parameters are only used when they are defined by crystal symmetry. An approximate (isotropic) treatment of cell e.s.d.'s is used for estimating e.s.d.'s involving l.s. planes.
Refinement. Refinement of *F*^2^ against ALL reflections. The weighted *R*-factor *wR* and goodness of fit *S* are based on *F*^2^, conventional *R*-factors *R* are based on *F*, with *F* set to zero for negative *F*^2^. The threshold expression of *F*^2^ > σ(*F*^2^) is used only for calculating *R*-factors(gt) *etc*. and is not relevant to the choice of reflections for refinement. *R*-factors based on *F*^2^ are statistically about twice as large as those based on *F*, and *R*- factors based on ALL data will be even larger.

### Fractional atomic coordinates and isotropic or equivalent isotropic displacement parameters (Å^2^)

**Table:**

	*x*	*y*	*z*	*U*_iso_*/*U*_eq_	Occ. (<1)
N1	0.4824 (3)	0.15042 (16)	0.05037 (19)	0.0661 (6)	
N2	1.2014 (4)	0.3925 (2)	0.0621 (2)	0.1031 (10)	
O1	0.3420 (3)	0.10682 (14)	0.08908 (15)	0.0762 (6)	
O2	0.6803 (3)	0.21500 (17)	−0.08246 (16)	0.1077 (9)	
H2	0.5962	0.1874	−0.0647	0.162*	
C1	0.2185 (4)	0.0666 (2)	0.0010 (2)	0.0731 (8)	
H1A	0.2763	0.0180	−0.0354	0.088*	
H1B	0.1725	0.1171	−0.0505	0.088*	
C2	0.0702 (3)	0.0215 (2)	0.0445 (2)	0.0721 (8)	
H2A	0.0162	0.0703	0.0828	0.086*	
H2B	0.1178	−0.0291	0.0954	0.086*	
C3	0.5929 (4)	0.1909 (2)	0.1266 (2)	0.0640 (8)	
H3	0.5727	0.1877	0.1965	0.077*	
C4	0.7474 (3)	0.24132 (19)	0.1074 (2)	0.0578 (7)	
C5	0.7878 (4)	0.2523 (2)	0.0062 (2)	0.0638 (7)	
C6	0.9367 (4)	0.3015 (2)	−0.0082 (2)	0.0736 (9)	
H6	0.9585	0.3077	−0.0776	0.088*	
C7	1.0547 (4)	0.3419 (2)	0.0766 (2)	0.0765 (9)	
C8	1.0136 (4)	0.3313 (3)	0.1787 (2)	0.1091 (14)	
H8	1.0893	0.3572	0.2387	0.131*	
C9	0.8640 (4)	0.2835 (3)	0.1913 (2)	0.0949 (11)	
H9	0.8397	0.2792	0.2602	0.114*	
C10	1.2063 (14)	0.4298 (8)	−0.0475 (9)	0.086 (3)	0.546 (16)
H10A	1.2705	0.4908	−0.0404	0.104*	0.546 (16)
H10B	1.0857	0.4428	−0.0852	0.104*	0.546 (16)
C11	1.2923 (12)	0.3612 (8)	−0.1148 (11)	0.117 (4)	0.546 (16)
H11A	1.4115	0.3474	−0.0779	0.175*	0.546 (16)
H11B	1.2946	0.3908	−0.1832	0.175*	0.546 (16)
H11C	1.2254	0.3020	−0.1261	0.175*	0.546 (16)
C12	1.3562 (12)	0.3955 (8)	0.1481 (10)	0.094 (4)	0.525 (13)
H12A	1.4652	0.3943	0.1205	0.112*	0.525 (13)
H12B	1.3569	0.3425	0.1986	0.112*	0.525 (13)
C13	1.3294 (17)	0.4919 (10)	0.1982 (10)	0.094 (4)	0.525 (13)
H13A	1.3161	0.5417	0.1441	0.141*	0.525 (13)
H13B	1.4304	0.5063	0.2542	0.141*	0.525 (13)
H13C	1.2245	0.4894	0.2284	0.141*	0.525 (13)
C10'	1.2900 (15)	0.3771 (9)	−0.0279 (11)	0.086 (4)	0.454 (16)
H10C	1.2714	0.3111	−0.0542	0.104*	0.454 (16)
H10D	1.4167	0.3880	−0.0051	0.104*	0.454 (16)
C11'	1.2124 (14)	0.4477 (10)	−0.1155 (12)	0.105 (4)	0.454 (16)
H11D	1.0856	0.4408	−0.1322	0.158*	0.454 (16)
H11E	1.2594	0.4346	−0.1788	0.158*	0.454 (16)
H11F	1.2427	0.5127	−0.0913	0.158*	0.454 (16)
C12'	1.298 (2)	0.4652 (12)	0.1485 (10)	0.083 (4)	0.475 (13)
H12C	1.3526	0.5158	0.1128	0.100*	0.475 (13)
H12D	1.2113	0.4956	0.1838	0.100*	0.475 (13)
C13'	1.4401 (12)	0.4176 (7)	0.2334 (9)	0.091 (4)	0.475 (13)
H13D	1.3899	0.3632	0.2640	0.136*	0.475 (13)
H13E	1.4846	0.4637	0.2891	0.136*	0.475 (13)
H13F	1.5355	0.3957	0.2007	0.136*	0.475 (13)

### Atomic displacement parameters (Å^2^)

**Table:**

	*U*^11^	*U*^22^	*U*^33^	*U*^12^	*U*^13^	*U*^23^
N1	0.0676 (15)	0.0703 (15)	0.0680 (15)	0.0002 (13)	0.0315 (13)	0.0105 (13)
N2	0.080 (2)	0.156 (3)	0.0764 (19)	−0.036 (2)	0.0222 (17)	0.008 (2)
O1	0.0714 (13)	0.0932 (15)	0.0707 (13)	−0.0087 (11)	0.0302 (11)	0.0124 (11)
O2	0.135 (2)	0.137 (2)	0.0643 (14)	−0.0630 (17)	0.0500 (13)	−0.0323 (13)
C1	0.070 (2)	0.082 (2)	0.0717 (19)	−0.0014 (17)	0.0248 (16)	0.0077 (16)
C2	0.0660 (19)	0.081 (2)	0.0742 (19)	0.0035 (16)	0.0250 (14)	0.0138 (16)
C3	0.0654 (19)	0.077 (2)	0.0546 (16)	0.0103 (16)	0.0241 (15)	0.0148 (15)
C4	0.0542 (16)	0.0727 (18)	0.0495 (15)	0.0095 (14)	0.0179 (13)	0.0136 (13)
C5	0.0772 (19)	0.0689 (18)	0.0508 (16)	−0.0059 (16)	0.0262 (15)	−0.0074 (14)
C6	0.087 (2)	0.086 (2)	0.0577 (17)	−0.0134 (18)	0.0392 (17)	−0.0018 (15)
C7	0.064 (2)	0.105 (2)	0.0617 (19)	−0.0059 (18)	0.0168 (16)	0.0116 (17)
C8	0.069 (2)	0.203 (4)	0.0511 (18)	−0.034 (2)	0.0026 (15)	0.017 (2)
C9	0.069 (2)	0.172 (3)	0.0444 (17)	−0.015 (2)	0.0130 (15)	0.0235 (19)
C10	0.078 (6)	0.103 (7)	0.082 (7)	−0.016 (5)	0.024 (5)	0.006 (6)
C11	0.118 (6)	0.125 (8)	0.124 (10)	0.000 (6)	0.063 (6)	0.014 (6)
C12	0.074 (6)	0.095 (7)	0.115 (10)	0.007 (5)	0.027 (7)	−0.001 (6)
C13	0.083 (7)	0.086 (7)	0.106 (9)	−0.006 (6)	0.003 (7)	−0.012 (7)
C10'	0.067 (6)	0.105 (8)	0.089 (9)	−0.004 (5)	0.020 (5)	0.012 (6)
C11'	0.099 (7)	0.119 (10)	0.096 (8)	0.006 (6)	0.016 (6)	0.022 (7)
C12'	0.068 (7)	0.102 (11)	0.079 (8)	−0.021 (7)	0.011 (7)	0.002 (7)
C13'	0.070 (6)	0.111 (7)	0.083 (7)	0.003 (5)	−0.004 (5)	−0.016 (5)

### Geometric parameters (Å, °)

**Table:**

N1—C3	1.278 (3)	C9—H9	0.9300
N1—O1	1.407 (2)	C10—C11	1.51 (2)
N2—C7	1.370 (4)	C10—H10A	0.9700
N2—C12	1.442 (12)	C10—H10B	0.9700
N2—C10'	1.456 (14)	C11—H11A	0.9600
N2—C10	1.486 (12)	C11—H11B	0.9600
N2—C12'	1.557 (13)	C11—H11C	0.9600
O1—C1	1.422 (3)	C12—C13	1.50 (2)
O2—C5	1.353 (3)	C12—H12A	0.9700
O2—H2	0.8200	C12—H12B	0.9700
C1—C2	1.497 (3)	C13—H13A	0.9600
C1—H1A	0.9700	C13—H13B	0.9600
C1—H1B	0.9700	C13—H13C	0.9600
C2—C2^i^	1.515 (5)	C10'—C11'	1.50 (3)
C2—H2A	0.9700	C10'—H10C	0.9700
C2—H2B	0.9700	C10'—H10D	0.9700
C3—C4	1.438 (3)	C11'—H11D	0.9600
C3—H3	0.9300	C11'—H11E	0.9600
C4—C9	1.372 (4)	C11'—H11F	0.9600
C4—C5	1.386 (3)	C12'—C13'	1.52 (2)
C5—C6	1.374 (4)	C12'—H12C	0.9700
C6—C7	1.376 (4)	C12'—H12D	0.9700
C6—H6	0.9300	C13'—H13D	0.9600
C7—C8	1.398 (4)	C13'—H13E	0.9600
C8—C9	1.363 (4)	C13'—H13F	0.9600
C8—H8	0.9300		
			
C3—N1—O1	111.3 (2)	C9—C8—H8	119.6
C7—N2—C12	119.3 (4)	C7—C8—H8	119.6
C7—N2—C10'	124.0 (5)	C8—C9—C4	123.4 (3)
C12—N2—C10'	98.6 (6)	C8—C9—H9	118.3
C7—N2—C10	118.5 (4)	C4—C9—H9	118.3
C12—N2—C10	121.4 (5)	N2—C10—C11	114.0 (12)
C10'—N2—C10	38.4 (4)	N2—C10—H10A	108.7
C7—N2—C12'	121.5 (5)	C11—C10—H10A	108.7
C12—N2—C12'	41.1 (6)	N2—C10—H10B	108.7
C10'—N2—C12'	114.2 (6)	C11—C10—H10B	108.7
C10—N2—C12'	109.6 (6)	H10A—C10—H10B	107.6
N1—O1—C1	109.36 (19)	N2—C12—C13	100.4 (9)
C5—O2—H2	109.5	N2—C12—H12A	111.7
O1—C1—C2	108.2 (2)	C13—C12—H12A	111.7
O1—C1—H1A	110.1	N2—C12—H12B	111.7
C2—C1—H1A	110.1	C13—C12—H12B	111.7
O1—C1—H1B	110.1	H12A—C12—H12B	109.5
C2—C1—H1B	110.1	N2—C10'—C11'	107.7 (13)
H1A—C1—H1B	108.4	N2—C10'—H10C	110.2
C1—C2—C2^i^	111.8 (3)	C11'—C10'—H10C	110.2
C1—C2—H2A	109.3	N2—C10'—H10D	110.2
C2^i^—C2—H2A	109.3	C11'—C10'—H10D	110.2
C1—C2—H2B	109.3	H10C—C10'—H10D	108.5
C2^i^—C2—H2B	109.3	C10'—C11'—H11D	109.5
H2A—C2—H2B	107.9	C10'—C11'—H11E	109.5
N1—C3—C4	122.0 (2)	H11D—C11'—H11E	109.5
N1—C3—H3	119.0	C10'—C11'—H11F	109.5
C4—C3—H3	119.0	H11D—C11'—H11F	109.5
C9—C4—C5	115.8 (2)	H11E—C11'—H11F	109.5
C9—C4—C3	120.5 (2)	C13'—C12'—N2	113.2 (13)
C5—C4—C3	123.7 (3)	C13'—C12'—H12C	108.9
O2—C5—C6	117.6 (2)	N2—C12'—H12C	108.9
O2—C5—C4	120.8 (2)	C13'—C12'—H12D	108.9
C6—C5—C4	121.6 (3)	N2—C12'—H12D	108.9
C5—C6—C7	122.1 (3)	H12C—C12'—H12D	107.8
C5—C6—H6	118.9	C12'—C13'—H13D	109.5
C7—C6—H6	118.9	C12'—C13'—H13E	109.5
N2—C7—C6	122.1 (3)	H13D—C13'—H13E	109.5
N2—C7—C8	121.5 (3)	C12'—C13'—H13F	109.5
C6—C7—C8	116.3 (3)	H13D—C13'—H13F	109.5
C9—C8—C7	120.8 (3)	H13E—C13'—H13F	109.5
			
C3—N1—O1—C1	177.3 (2)	N2—C7—C8—C9	177.8 (4)
N1—O1—C1—C2	−179.6 (2)	C6—C7—C8—C9	−0.1 (5)
O1—C1—C2—C2^i^	178.8 (3)	C7—C8—C9—C4	1.4 (6)
O1—N1—C3—C4	−179.4 (2)	C5—C4—C9—C8	−1.7 (5)
N1—C3—C4—C9	−179.6 (3)	C3—C4—C9—C8	179.3 (3)
N1—C3—C4—C5	1.5 (4)	C7—N2—C10—C11	−92.1 (8)
C9—C4—C5—O2	−178.9 (3)	C12—N2—C10—C11	78.4 (9)
C3—C4—C5—O2	0.1 (4)	C10'—N2—C10—C11	17.7 (8)
C9—C4—C5—C6	0.7 (4)	C12'—N2—C10—C11	122.3 (9)
C3—C4—C5—C6	179.7 (3)	C7—N2—C12—C13	−97.9 (8)
O2—C5—C6—C7	−179.8 (3)	C10'—N2—C12—C13	125.0 (8)
C4—C5—C6—C7	0.6 (5)	C10—N2—C12—C13	91.8 (9)
C12—N2—C7—C6	−152.3 (6)	C12'—N2—C12—C13	7.7 (11)
C10'—N2—C7—C6	−26.5 (8)	C7—N2—C10'—C11'	93.2 (9)
C10—N2—C7—C6	18.3 (7)	C12—N2—C10'—C11'	−132.5 (8)
C12'—N2—C7—C6	159.7 (8)	C10—N2—C10'—C11'	−1.3 (8)
C12—N2—C7—C8	29.9 (7)	C12'—N2—C10'—C11'	−92.7 (10)
C10'—N2—C7—C8	155.7 (7)	C7—N2—C12'—C13'	88.0 (10)
C10—N2—C7—C8	−159.5 (6)	C12—N2—C12'—C13'	−11.8 (7)
C12'—N2—C7—C8	−18.1 (9)	C10'—N2—C12'—C13'	−86.3 (10)
C5—C6—C7—N2	−178.7 (3)	C10—N2—C12'—C13'	−127.6 (9)
C5—C6—C7—C8	−0.8 (5)		

Symmetry codes: (i) −*x*, −*y*, −*z*.

### Hydrogen-bond geometry (Å, °)

**Table:**

*D*—H···*A*	*D*—H	H···*A*	*D*···*A*	*D*—H···*A*
O2—H2···N1	0.82	1.91	2.639 (2)	147
